# Myelitis of the Dorsal Region, Accompanying Pregnancy

**Published:** 1886-09

**Authors:** Edward B. Angell


THE BUFFALO Medical and Surgical Journal

VOL. XXVI. SEPTEMBER, 1886. NO. 2.

### Original Communications.

### MYELITIS OF THE DORSAL REGION, ACCOMPANYING PREGNANCY.

BY EDWARD B. ANGELL, M. D.

(Read before the recent meeting of the Central New York Medical Association, at Rochester, May 1886.)

On the 4th of March last, I was called, in consultation, to see Mrs. H., of this city. Inasmuch as the disease, doubtless myelitis of the dorsal region, presented many anomalous features, which, with marked hysterical symptoms, masked the organic malady, while a pre-existing pregnancy developed, unretarded, till death, the case has been deemed of sufficient interest to be presented to the Association. The previous history of the case, gathered from various sources, was as follows:

The patient, thirty years of age, of a marked neurotic temperament, was a French Canadian by birth. She had married ten years ago, and gave birth to a healthy child one year later. Since then she has had two miscarriages, at three months. Her menstrual history was in no way significant. Her family history was good, and her own health always satisfactory, though her temper was strong and obstinate. Previous to the paralytic trouble her health had been very good. Her last menstruation occurred October 18th, and five weeks later, while daily expecting the usual recurrence of the menstrual flow, the paralytic symptoms appeared, and developed with considerable rapidity. Regarding any immediate cause for the trouble, the history is not so clear. So far as could be ascertained, the history, on either side, with reference to syphilitic trouble, was perfectly clear. The patient herself asserted that she had taken a hard cold, and shortly afterward, was affected with a peculiar numbness in the right leg. For one week preceding the onset of the paralytic symptoms she had experienced dull, aching pain in the legs, more especially marked in the feet. During this period the legs were weak and heavy, rendering locomotion difficult and slow. The dull ache, beginning in the legs, gradually extended during the week and involved the trunk as high up as the umbilicus. Notwithstanding this growing weariness she continued her house-work, till one evening, after an unusually hard days' work, a nervous shock seemed to precipitate matters. It may be well to add, just here, that I learned, through a very reliable source, the patient had had a severe tussel with an intoxicated father-in-law, and received several hard blows upon the back, in the neighborhood of the sacrum. But, at the time of my examination, no trace of such injury was evident, nor could I learn through the other attending physicians that these blows had left any external signs. During the night succeeding the hard day's work she awoke to find she had lost sensation in the right leg, though suffering severe neuralgic pain along the course of the siatic nerve. During the following week motor power remained, though greatly restricted. Then motor paralysis invaded the right foot, and, gradually extending upward, involved the whole leg in the course of the following two weeks. The left leg was affected in a similar manner, and by the middle of December the motor paralysis had implicated the trunk up to a line on a level with the umbilicus. About the middle of November, she noticed loss of sensation in the bladder, rectum and vagina, followed, a week or so later, by entire motor palsy of these organs. Her bowels required the aid of powerful laxatives to secure action, and the catheter was used regularly, from this time, until her death. By reason of this loss of sensation she was wholly unconscious of the passage of feces, or of the contact of the catheter with the urethra. But, curiously, up to within a short time of her death, she recognized the need of an evacuation by a sense of abdominal fullness, and the placing her upon a commode was quite sufficient to establish the mechanism of defecation. This partial reflex control over the corresponding function of urination, however, did not exist at any time, retention being only attended with reflex pain in the limbs, while the early and persistent use of the catheter never allowed it to become incontinent.

About the beginning of December she was attacked with extreme nausea and vomiting, with little ability to retain nourishment. So serious did this trouble become, that, for some time, her life was despaired of. But, in the course of a month or six weeks, she recovered from that disturbance and thereafter was able to eat and digest her food satisfactorily. Whether this was an aggravated form of morning sickness, or, in part, dependent upon the spinal malady, one could scarcely venture an opinion.

From the time of the complete establishment of the paralysis, up to the date of my examination, very little change was noted, other than the development of a very ugly, irrascible temper, of a marked hysterical type; and the limit of sensation and power was then two inches above the umbilicus.

During the first week of March a careful examination elicited the following features: The patient appeared well nourished, of good color, and, with the exceptions noted above, her vital functions were well performed. The heart, lungs and liver were normal, the pulse rate was 80 and excellent in character, while the respiratory rate was about

20 and regular. There was no fever. The usual signs indicated the existence of pregnancy, and the abdomen was so encroached upon by the gravid uterus that its thorough exploration was impossible. The expression of the countenance was somewhat anxious, and the temper unstable. She evidently suffered great pain, though it seemed uncertain whether it was subjective or real. Certainly the expression of its severity was very greatly influenced by the circumstances of the moment, very much increased, on the one hand, by the presence of the physician or sympathizing friends, and, on the other, greatly modified by diverting the attention. Inasmuch as I cannot overdraw the emotional features in a truthful portrayal of this case, I dwell a moment upon this phase of her character, as it was displayed from time to time. She was most exacting in her demand for sympathy, and very jealous if it was at all denied her. At times she treated her nurse and the family most shamefully, abusing them outrageously, while an occasional attack of hysteria, attended with sobbing and screaming, tearing her hair and clothing, hurling any article at hand about the room, and other displays of the worst features of uncontrolable emotion, gave striking character to the picture. Yet, in the very worst attack, a small hypodermatic injection of morphia, or the first whiff of ether, would quiet her excitement so easily as, apparently, to determine, beyond doubt, the nature of her malady. So completely did this aggravated hysterical condition mask the deeper symptoms, that every physician—and several were consulted during the progress of the disease—was misled to the conclusion that it must be hysterical paraplegia. Toward forming this conclusion the peculiar correlation of the objective symptoms assisted materially. Let us now examine them.

Voluntary control of the body, below a well defined line about the trunk, was absolutely cut off, there being complete motor palsy of the legs and feet. The rectum and bladder were implicated in the paralysis, as has been noted above, requiring the use of cathartics and the catheter. This line of demarkation was very definite in front, about an inch below the ensiform cartilage, and somewhat lower—perhaps by an inch and a half—behind, though the left half of this girdle was rather lower than the right, Anaesthesia over the same area was also complete, every inch of the skin, as we l as the mucous surfaces of the rectum and vagina, being devoid of sensation of any sort, there being no reaction to touch, to heat, to cold, or to pain, however severe. In testing analgesia the wire brush was used, with both galvanic and faradic currents. Up to the line, above described the strongest currents caused no pain, while half an inch higher, mild currents were most severe. I said loss of sensation over the same area was complete; I should except the dorso-lumbar region of the spine, where there was considerable hyperaesthesia, together with marked muscle rigidity. The electrical tests indicated the absence also of the muscle sense. But concussion of the surfaces of the joints of the legs did produce some trace of sensation, though the statements of the patient on this point cannot be considered reliable. Furthermore there was no bleeding from pin pricks in the affected region. The reflexes were exaggerated, the skin reflexes being very marked up to the epigastric, which was somewhat deficient. Application of the electric brush caused curling of the toes, when applied to the feet, and muscle spasm when used elsewhere. The knee-jerk was excessive, more so on the right side; the right leg also afforded a good ankle clonus, thus indicating the integrity, with overaction, of the lumbar reflex loop. The electrical examination of the leg-muscles showed no reaction of degeneration, the only change being that the reaction to the faradic current was rather retarded, though not deficient in force. The galvanic reaction was slight. There was no wasting of the muscles involved, neither was there any spasmodic rigidity of the limbs, other than a slight stiffness under passive motion. At times there was some spasmodic twitching of the legs, more evident during the application of electricity. There were no bed sores at the time, and no apparent alteration in the nutrition of the skin or nails. There was, however, considerable anasarca of the fore legs and feet, the ankles showing marked pitting on pressure. Examination of urine, at various times, gave these results invariably: There action was acid; the sp. gr. high, from 1025 to 1030 or 1035; the color, brownish red; considerable deposit on standing; no albumen; no sugar; no casts; strong odor; considerable pus; some mucous cells and numerous crystals of lime oxalate. At no time did it seem to be alkaline, when freshly drawn. At the time of examination, the past history of the case could not be well made out, and the singular combination of symptoms, the absence of tropic changes, usually so marked in myelitis, the preservation of the lumbar reflex loop, the pressence of the normal electrical reactions, the nearly normal character of the urine, and the supposed sudden onset of the disease in all its gravity, after a nervous shock, emphasized by the very remarkable play of hysterical phenomena, almost forced the erroneous diagnosis ot hysterical paraplegia. This conclusion, however, was shaken by the progress of the case; and its fatal termination, together with points in the history, previously undeveloped, established, as well as could be established without an examination of the cord, the nature of the malady. The treatment offers nothing of interest, unless a confession of failure and disappointment is worthy of note. Under the use of the faradic wire brush and large doses of ergot, however, there was some lessening of the area of anaesthesia at first, the limit of sensation being lowered some two inches.

Nearly two weeks later, or on the 17th of March, there was noticed some abrasions of the skin, on the inner side of the buttocks, though trivial and yielding to appropriate treatment; there was also noticed, at the same time a forming bed-sore, on the right heel. The most puzzling alteration, however, at this time, was a total loss of the skin and knee reflexes, so marked at the earlier examination, together with entire absence of the muscle twitching. There was also some bleeding in the feet from pin points. There was increased pain, requiring the use of heavy doses of morphia for relief. On the 20th the back was easier, but a burning sensation had developed in the legs and feet, with increasing local pain. On the afternoon of the 21st the respiration seemed affected, more shallow, and somewhat more frequent than normal, the thoracic muscles acting with difficulty. There was some fever, 103°, and the usual symptoms of static congestion of the lungs. But this condition was considerably relieved during the next few days, only to be followed by a much graver attack of respiratory paralysis on the 28th, an attack which baffled all remedial measures, and which resulted in death from respiratory failure late on the following day. A thorough post-mortem examination was much desired, but permission only to open the abdomen and remove the fœtus was secured. This partial examination showed the abdominal organs in normal condition, a large amount of fat on the abdomen, the muscular tissues thinned, and a gravid uterus containing a well-developed fœtus, at about the sixth month of gestation, and, apparently, only very recently dead. Lacking the confirmation of a post-mortem examination of the cord, the writer can only suggest that the disease probably was a cortical myelitis, located at about the middle of the dorsal region, affecting mainly the post-sensory nerve roots and the lateral conducting tracts of white matter, and invading the gray, motor region of the cord only at a very late period in its course, when rapid extension downward implicated the anterior motor cells of the lumbar region, with consequent loss of the lumbar reflexes, while rapid extension upward involved the respiratory muscles, causing the fatal termination.

